# Advances in Bcl-xL Research 2.0

**DOI:** 10.3390/ijms24119484

**Published:** 2023-05-30

**Authors:** Joann Kervadec, Muriel Priault

**Affiliations:** Institut de Biochimie et de Génétique Cellulaires, Université de Bordeaux, CNRS, UMR 5095, 33077 Bordeaux, France

Apoptosis is a form of programmed cell death that is highly conserved in metazoan organisms, where it ensures the proper development and homeostasis of tissues. It is regulated by the multigenic families of proteins (e.g., death receptors, caspases, and Bcl-2 proteins) which actin the extrinsic and/or in the intrinsic pathway. In the latter, the Bcl-2 family of proteins is dedicated to controlling mitochondrial permeability and inducing the release into the cytosol of apoptogenic factors require to accelerate and amplify signal transduction, resulting in orderly cell death. The Bcl-2 family is composed of small globular proteins that are essentially organized in alpha helices. They share homology motifs called BH (Bcl-2 Homology), but are functionally divided into three subgroups: pro-survival proteins (including Bcl-2 and Bcl-xL) which bind and inhibit pro-death proteins (including Bax and Bak), and BH3-only proteins (like Bad, Bid, and Bim…) which modulate the interactions between the former two. This Special Issue collates reviews and articles illustrating recent advances in Bcl-xL research, as this year celebrates the 30th anniversary of its discovery.

Since its discovery in 1993 [1] and in the past three decades, Bcl-xL has been described as a protein with a wide expression profile in terms of time (it is not restricted to a developmental stage) and in tissues (its expression is ubiquitous, both in solid and liquid tissues). A detailed overview of Bcl-xL transcriptional regulation is presented in this Special Issue by Lucianò et al. [2], and by Morales-Martínez and Vega [3]. The fact that Bcl-xL specializes in the survival of two particular cell types, neurons during development and platelets in mature organisms, is emphasized herein by two reviews. Josefsson et al. discuss how platelets, the small anucleated cells operating in hemostasis and blood clotting, depend on Bcl-xL for their survival and on Bak for their death. Notably, this review reiterates that, despite the intense investigations that have been carried out, the mechanism disrupting the balance between Bak and Bcl-xL and leading to platelet apoptosis remains unknown [4].

Bas et al. review the role of Bcl-xL in neuronal function and development. Notably, their work emphasizes a research area that has become prominent since approximately 2010, in which non-canonical functions of Bcl-2 proteins are under scrutiny. This review discusses the particular ability of Bcl-xL to regulate mitochondrial dynamics through its interaction with DRP1, and its ability to contribute to calcium homeostasis via binding to VDAC1. These non-canonical functions are presented in light of the potential role of Bcl-xL as an activator of mitochondrial metabolism in neurons, as these cells have high energy demands for the dynamic regulation of synapse formation and destruction [5] (Figure 1).

Anti-apoptotic Bcl-2 proteins constitute a class of oncogenes that specialize in cell survival rather than cell proliferation. Dysregulations of these proteins have been related to a wide variety of diseases including cancer, neurodegenerative diseases and hemopathies, as well as susceptibility to viral infection. This Special Issue presents a number of articles dealing with the pathological aspects of Bcl-xL. Bas et al. discuss how impaired Bcl-xL activity correlates to different neuronal diseases such as Parkinson’s disease, amyotrophic lateral sclerosis, and spinal cord muscular atrophy [5]. Lucianò et al. review the role Bcl-xL plays in the pathobiology of cutaneous melanoma, a deadly form of skin cancer. They discuss the evidence that increased expression of Bcl-xL correlates to progression from primary to metastatic melanoma. This review presents other examples of the non-canonical functions of Bcl-xL, notably cell migration and invasion in melanoma models, and how this connects Bcl-xL to neoangiogenesis signaling pathways [2].

Dysregulations of Bcl-xL in blood-related diseases are the focus of two reviews: Josefsson et al. explain how the upregulation of Bcl-xL expression in a hematopoietic stem cell following the acquisition of somatic mutations translates into the onset of diseases of abnormal platelet numbers [4]; Morales-Martínez and Vega present bio-informatic analyses of the expression of Bcl-xL in hematological malignancies and explore the possibility of establishing Bcl-xL as marker of prognosis [3].

Finally, Wyżewski et al. review the complex interplay between viruses, Bcl-xL expression and the outcomes both for the survival of host cells and for promoting viral infections. Indeed, depending on the virus and the circumstances, Bcl-xL expression may either be upregulated or downregulated, with opposing outcomes for the infected cell [6].

Considering the wide diversity of diseases correlated to Bcl-xL dysregulation, it is only logical to infer its potential role as a therapeutic target. Several reviews of this Special Issue emphasize the role of Bcl-xL in resistance to chemotherapy and in the aggressiveness of cancers of different origins where this oncogene is over-expressed. Despite the poor characterization of the molecular mechanism(s) at play in Bcl-xL-related chemo-resistance, valuable and useful information has been revealed throughout the last decade through the refined knowledge of anti-apoptotic Bcl-2 protein structures. The design of small molecules mimicking the action of the BH3-only proteins (BH3 mimetics) that are able to abrogate the function of these oncogenes has introduced wide-ranging prospects in clinics. The targeting of anti-apoptotic Bcl-2 proteins in preclinical studies is reviewed here in the context of melanoma [2] and hematological malignancies [3] 2022). Zhang et al. present research exploring the response of 19 colorectal cancer cell lines to BH3 mimetics; the combined inhibition of Mcl-1 and Bcl-xL proves to be the most effective treatment. Furthermore, KRAS/BRAF mutations are suggested to be negative factors in predicting the sensitivity of CRC cell lines to BH3 mimetic treatment [7].

Rahman et al. discuss the mechanisms by which different classes of flavonoids may modulate Bcl-2, and to a lesser extent Bcl-xL, in the P53 signaling pathway, as well as their potential use in cancer therapy [8]. 

Finally, Tyagi et al. present a research article in which molecular dynamics simulations are used to study the conformation of Bcl-xL anchored into the membrane, which exhibits a significant degree of structural remodeling of the globular head. The conformational dynamics of Bcl-xL are explored using a protein with all aspartic and glutamic acid and histidine sidechains protonated or deprotonated as this modifies the interaction of Bcl-xL with membranes; the effect of the mitochondrial lipid cardiolipin is also analyzed due to the postulation that it generates hotspots for apoptotic regulation [9]. This work illustrates how considering molecular dynamics is useful for obtaining structural insights into the factors acting on conformational modifications; here, both the protonation of membrane-anchored Bcl-xL and cardiolipin are proposed to be required for the exposure of the BH3-binding groove.

Overall, Bcl-xL is involved in numerous physiological processes as well as pathological ones, both through its canonical and non-canonical functions. Many teams agree that it is a relevant therapeutic target. However, the challenge consists in our ability to inhibit pathological processes but spare the physiological ones. The key to this challenge may be revealed through structural studies, as illustrated by the advance of BH3 mimetics. These studies potentially contain the major breakthroughs of research in this field (though these are not exhaustively explained yet), which may result in clinical applications that are beneficial to patients.

## Figures and Tables

**Figure 1:**
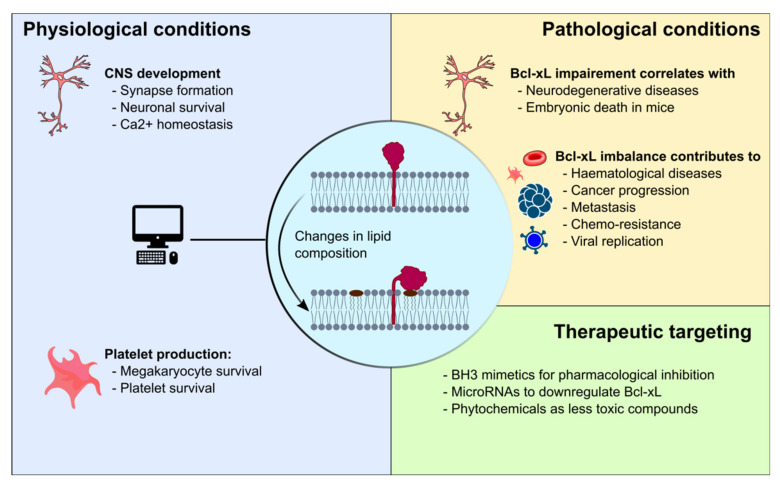
Involvement of Bcl-xL in different physiopathological processes.
